# Molecular Dissection of the Regenerative Capacity of Forest Tree Species: Special Focus on Conifers

**DOI:** 10.3389/fpls.2018.01943

**Published:** 2019-01-09

**Authors:** Carmen Díaz-Sala

**Affiliations:** Department of Life Sciences, University of Alcalá, Alcalá de Henares, Spain

**Keywords:** somatic embryogenesis, adventitious roots, adventitious shoots, auxin, stem cells, cell wall, cytoskeleton

## Abstract

Somatic embryogenesis (SE) and organogenesis have become leading biotechnologies for forest tree improvement and the implementation of multi-varietal forestry. Despite major advances in clonal propagation using these technologies, many forest tree species, such as conifers, show a low regeneration capacity. Developmental factors such as genotype, the type and age of the explant or tissue, and the age and maturity of the mother tree are limiting factors for the success of propagation programs. This review summarizes recent research on the molecular pathways involved in the regulation of key steps in SE and organogenesis of forest tree species, mainly conifers. The interaction between auxin and stress conditions, the induction of cell identity regulators and the role of cell wall remodeling are reviewed. This information is essential to develop tools and strategies to improve clonal propagation programs for forest tree species.

## Introduction

Recalcitrance to somatic embryogenesis (SE), adventitious rooting (AR) from stem cuttings or adventitious shoot (AS) formation is a major limitation for the clonal propagation/micropropagation of elite trees in many woody species, especially forest tree species. Several reviews on the state-of-the-art of the application of these techniques as well as the impacts of recalcitrance and tree domestication on forestry have been published (Bonga et al., 2010; Lelu-Walter et al., 2013; Bonga, 2015). There are several major factors that restrict the vegetative propagation response, including genotype within species, tissue type, time of excision, and the maturity level of the mother tree. In addition, the failure of induced somatic embryos to germinate or mature can reinforce recalcitrance when SE is used for the mass clonal propagation of selected genotypes. Bonga et al. (2010) proposed several possible causes and potential solutions for recalcitrance. In addition, many genes associated with the induction of SE and/or organogenesis have been described, mostly in model species, during the last decade (Ikeuchi et al., 2016). However, knowledge of the molecular and cellular mechanisms involved in recalcitrance of forest tree species, which is expressed under a wide range of genetic and physiological conditions, is still required to define pathways that may be relevant to solve problems related to differences in genotypes, tissues, time of excision, or age.

This review summarizes the results of recent studies on the molecular and cellular mechanisms involved in the regeneration capacity of forest tree species by SE, AR, or AS formation, with special focus on conifers. The interaction between auxin and stress conditions, the induction of cell identity regulators and the role of cell wall remodeling are mainly reviewed. We have focused on the early stages of regeneration, when the transition from a somatic to an embryonic or meristematic cell occurs, in an attempt to highlight factors or common pathways that may be relevant for the control of the recalcitrance.

## Plant Growth Regulators and Stress-Regulated Pathways

The induction of SE, AR, or AS formation is a multi-factorial developmental process that includes the effects of hormones and chemical and physical factors. Such factors include the composition of media, growth conditions, stress associated with wounding (excision) or the culture environment, and other stress factors (Karami and Saidi, 2010). It has been proposed that interactions among stress signaling, hormones, and developmental regulators result in the acquisition of embryogenic/morphogenic competence by direct or indirect cellular reprogramming of somatic cells (Fehér, 2015).

The conditions and requirements for the induction of SE, AR, or AS formation vary within and among forest tree species; however, some common pathways can be identified. The most obvious is the absolute requirement for auxin and polar auxin transport during the initiation stage. Plant growth regulators play a central role in both the initiation of pro-embryogenic masses (PEMs) and the differentiation and maturation of somatic embryos. In forest tree species, initiation and proliferation of embryogenic masses (EMs) are induced in the presence of auxin and cytokinin. Withdrawal of these plant growth regulators results in the differentiation of somatic embryos from PEMs. Embryos are subsequently transferred to medium supplemented with abscisic acid (ABA) to induce somatic embryo maturation.

Businge et al. (2012) focused on the role of auxin in the induction and proliferation of PEMs and EMs, and speculated that the presence of auxin at the proliferation and embryo differentiation stages was essential for embryos to develop to the maturation phase. This was indicated by the presence of tryptophan, a precursor of indol-3-acetic acid (IAA) biosynthesis, only in Norway spruce cell lines that formed mature somatic embryos. Endogenous IAA levels have been shown to be relatively higher during proliferation of EMs and somatic embryos of Norway spruce and *Abies alba* (Hakman et al., 2009; Vondráková et al., 2011). Although a decrease in IAA levels during embryo maturation has been reported for Norway spruce (Hakman et al., 2009), high levels of endogenous IAA induced by exogenous auxin in the proliferation medium resulted in increased frequency of maturation in embryogenic cultures of *A. alba* (Vondráková et al., 2011).

The maintenance of auxin transport and further reduction of local auxin gradients are also important for the induction of pro-embryos from EMs (Find et al., 2002; Larsson et al., 2008; Palovaara et al., 2010; Abrahamsson et al., 2011). Rodríguez-Sanz et al. (2014) described an increase in endogenous IAA levels during the initiation of PEMs from both microspore and zygotic embryos of *Quercus suber*. Inhibition of polar auxin transport by N-1-naphthylphthalamic acid (NPA) has been shown to negatively affect embryogenesis initiation and development. This indicates that high levels of endogenous IAA and polar auxin transport are required for resuming proliferation and activating the embryogenic program during cell reprogramming leading to SE. Similar results have been described during SE from adult leaf tissues of *Quercus alba* (Corredoira et al., 2017). Cells of the PEMs, which were located at the periphery and emerging from the leaf explants, showed intense IAA immunofluorescence labeling, whereas no IAA signal was detected in non-embryogenic cells adjacent to the intensely labeled PEMs. Labeling was localized in the cytoplasm of the EMs. Every cell in somatic embryos at early stages of development also displayed intense IAA immunofluorescence in the cytoplasm. These results indicated that high endogenous auxin levels may be involved in the activation of proliferation during reprogramming of adult cells leading to SE.

In contrast, low endogenous auxin levels at specific stages of somatic embryo development can facilitate the acquisition of competence for SE in forest tree species. Zhou et al. (2017) found that endogenous auxin levels at the cleavage stage were associated with somatic embryo initiation and with genotype-dependent competence to form somatic embryos in Chinese fir. Cleaved embryos contained the lowest levels of IAA prior to the emergence of the dominant embryos, and genotypes showing high competence for somatic embryo induction exhibited lower IAA levels at the cleavage stage.

Increased auxin maxima and asymmetrical auxin distribution at the cambial and rooting cells were observed in rooting-competent cuttings from *Pinus radiata* after excision (Abarca et al., 2014). Adventitious rooting and the formation of auxin gradients were inhibited in rooting-competent cuttings in the presence of NPA, indicating that polar auxin transport was required for auxin accumulation at the base of the cutting (Díaz-Sala et al., 1996) and for auxin flow and asymmetric localization in rooting cells (Abarca et al., 2014; Brunoni et al., 2014). Rooting recalcitrance in a difficult-to-root species of *Eucalyptus* was found to be associated with changes in auxin concentration and sensitivity during the early age-related decline in rooting competence. A relatively lower auxin content in the cambium and/or repression of polar auxin transport proteins (among other proteins) have been shown to reduce the level and distribution of auxin in rooting cells (Ruedell et al., 2013, 2015; De Almeida et al., 2015; Aumond et al., 2017). Similar results have been described for chestnut. Vielba et al. (2016) suggested a role of CsGH3-1 in regulating rooting cell auxin homeostasis associated with maturation. However, neither increasing auxin concentrations nor auxin localization at the rooting site was sufficient to overcome the rooting recalcitrance of mature pine (Sánchez et al., 2007; Ricci et al., 2008), indicating that other cellular factors are involved.

Stevens et al. (2018) used laser capture microdissection to perform site-specific analysis of auxin-related gene expression in rooting and non-rooting progenitor cells of black walnut. They found that the expression of *PIN FORMED (PIN)3* and *PIN7* was only marginally influenced by time after induction, physiological age, or auxin treatments. However, the expression of *AUXIN RESPONSE FACTOR (ARF)6* and *ARF8*, which promote AR in model species, increased only in rooting-competent parenchyma cells. In addition, expression of *ARF17*, which inhibits rooting, decreased concomitantly with the increase in *ARF6* and *ARF8* expression. Auxin was required for localizing changes in gene expression. Recalcitrant mature cuttings showed a diffuse and uniform mRNA signal among all tissue types. The authors concluded that recalcitrance of black walnut to AR resulted from the failure of rooting-competent cells to perceive molecular signals initiated by auxin in mature cuttings. This confirmed the results of earlier studies on AR in conifers, demonstrating that auxin acts as an unspecific signal of preset cell reaction patterns (Díaz-Sala et al., 1996; Goldfarb et al., 1998; Greenwood et al., 2001).

The role of specific auxin-signaling pathways in the early stages of AR in *Populus* has been demonstrated by Shu et al. (2018). They found that the auxin receptor TIR1 homolog correlated with auxin distribution at the early stages of adventitious root formation (Liu et al., 2014) and regulated AR through interacting with AUX/IAA28. Components of auxin and cytokinin signaling also showed significant differential expression during *in vitro* dedifferentiation and shoot regeneration of poplar; the major regulatory events occurred at the early stages of dedifferentiation. However, genes related to auxin signaling, such as those encoding auxin F-box receptors and several AUX/IAAs and ARFs showed differential expression, and were highly regulated during callus initiation as compared with the small number of genes related to cytokinin signaling (Bao et al., 2009). Changes in auxin metabolism and signal transduction, as well as gradient formation have been described during SE, AR, and AS formation in model species. Such findings have indicated that regulatory pathways are conserved between herbaceous and woody plants, and between angiosperm and gymnosperm tree species (Bellini et al., 2014; Brunoni et al., 2014).

An interaction between stress and hormonal pathways during the acquisition of regeneration competence in forest tree species has been described (Da Costa et al., 2013). The *in vitro* culture environment combined with certain stressors, such as temperature, was shown to induce somatic embryos in cultures of mature recalcitrant gymnosperms. However, they still failed to develop into mature somatic embryos, indicating that additional factors or developmental pathways are involved in embryo maturation (Park et al., 2010). The ability to control oxidative stress to prevent oxidative damage and maintain cell and protein structure appeared to be a key component for embryogenesis induction in larch (Liu et al., 2016). Treatments with hydrogen, an antioxidant molecule, increased the proportion of active pro-embryogenic cells in PEMs, and induced normal somatic embryos. This effect of the hydrogen treatment was linked to the expression of stress-related genes in larch PEMs, reduced reactive oxygen species (ROS) levels, and enhanced antioxidant enzyme activity. These findings indicated that increased rates of active PEMs and normal somatic embryos may be, at least partially, a result of reduced ROS levels after hydrogen treatment (Liu et al., 2016). Up-regulation of flavonoid biosynthesis genes and related transcription factors in the EMs of Norway spruce may have promoted embryo development by regulating polar auxin transport and the functions of housekeeping enzymes, in addition to the more general role of flavonoids in ROS scavenging and abiotic stress resistance (Reza et al., 2018).

The identification of stress-related genes during the acquisition of SE capacity and embryo development in conifers reinforces the importance of stress defense responses in forest tree species (Aquea and Arce-Johnson, 2008; Vestman et al., 2011; Businge et al., 2012; Zhou et al., 2014). A comparison of metabolic profiles between Norway spruce cell lines that were competent and non-competent for SE demonstrated the accumulation of metabolites involved in the stress response, such as 4-aminobutyric acid, pinitol, and inositol during embryo formation in competent lines. This accumulation was associated with normal embryo development. On the basis of the high and differential expression of the conifer-specific Dehydrin gene 8 (DHN1) in embryogenic explants, the authors speculated that the moderate stress response of the responsive genotype was indicative of an adaptive stress response that may be an important determinant of somatic embryo induction. Differential expression of the conifer-specific DHN1 may also indicate that fundamental differences exist in the molecular regulation of SE induction between conifers and angiosperms (Rutledge et al., 2017). Genes encoding heat shock proteins, glutathione S-transferases, and enzymes involved in the phenylpropanoid biosynthesis showed significant differential expression between embryonic-competent tissues and non-embryonic masses of three *Picea balfouriana* genotypes (Li Q. et al., 2014). Several stress-related proteins also showed increased abundance during the acquisition of embryogenic competence and during different stages leading to pro-embryogenic callus formation in oil palm. Such proteins included those involved in the oxidative stress response, such as superoxide dismutase, a putative secretory peroxidase, ascorbate peroxidase, and pathogenesis-related (PR) proteins (De Carvalho Silva et al., 2014).

Injury and loss of the root–shoot relationship after cutting or explant excision are the major sources of stress during induction of AR from cuttings. Other conditions, such as flooding or nutrient deprivation, also affect AR (Braun et al., 2005; Parent et al., 2011; Steffens and Rasmussen, 2016). The results of Da Costa et al. (2013) supported the hypothesis that AR from cuttings is comparable to a stress-induced reprogramming of shoot cell fate. Acclimation to stress could protect cuttings, but it reduced the extent of the wound response, which inhibited rooting (De Klerk et al., 2011; Santos-Macedo et al., 2012). Changes in the transcriptome during the first 24 h of the root induction process in *Populus* were likely associated with stress and wounding responses that occurred immediately upon harvesting of the cuttings (Ribeiro et al., 2016). A comparative analysis of the transcriptome and proteome among clones of hybrid larch with different rooting capacities indicated that late embryogenesis abundant (LEA) and PR proteins were highly expressed during the early stages of AR in clones with a high rooting capacity (Han et al., 2014). The authors concluded that both types of proteins may have important roles in AR by either positively regulating the response to various stresses and enhancing lignification, or by protecting the cut tissues from damage caused by bacteria and fungi soon after cutting, thus allowing normal AR to occur. Similar results were described during AR in *Robinia pseudoacacia* (Zhang et al., 2015). They found that antioxidant enzymes and PR proteins such as peroxidases, glucan endo-1,3-beta-glucosidase, phenylcoumarin benzylic ether reductase, alcohol dehydrogenase, and protein inhibitors were up-regulated during induction of adventitious root primordia to scavenge ROS and prevent oxidative damage and/or defend against pathogen invasion after wounding.

The high expression levels of proteins involved in the stress response indicates a general stress adaptation process that may be related to the fine regulation of auxin and stress signaling. In the case of SE, this stress adaptation process may be associated with the withdrawal of auxin during the transition among early developmental stages, and the scavenging of ROS when PEMs are exposed to a new environment, for example, when larch PEMs were moved to an ABA-containing hypertonic medium for maturation (Zhao et al., 2015). However, the responses of explants to SE or organogenesis are strongly dependent on age, position, timing, and genetic traits. Interactions among the stress response, age, and genotype have been reported for several forest tree species, and positive and negative correlations have been described (Park et al., 2010; Rutledge et al., 2013; Zhou et al., 2017). No differences in the stress response to wounding were detected during the maturation-related decrease in AR in loblolly pine (Díaz-Sala et al., 1996; Greenwood et al., 2001). In contrast, persistent and strong expression of defense genes encoding apoplastic class III peroxidases, cell wall invertases, and I20 serine protease inhibitors was detected during the SE induction process in SE-recalcitrant lines of white spruce (Rutledge et al., 2013).

Endogenous auxin balance and/or responses, as well as stress conditions inherent to explant excision and culture environment are of significant importance for induction and development of SE and organogenesis. Variations in the redox state of cells, caused by stress conditions, may modify auxin homeostasis by affecting auxin biosynthesis, transport or metabolism. At the same time, auxin may be involved in regulation of the stress response to prevent stress damage. The interaction between both factors depends on the species, developmental stage, or type, age and source of explants, and may be partially related to the variability of the response to inductive conditions.

## Stem Cell Regulators and Developmentally Regulated Pathways

The acquisition of competence and cellular reprogramming from a somatic cell to a totipotent or multipotent cell resulting in a meristem or an embryo is a major intermediate step during regeneration. Previous reports have described several stem cell regulators, some of them involved in auxin distribution or stress response, that participate in the induction of SE, AR, or AS formation.

Several genes encoding transcription factors showed high expression levels in highly responsive somatic genotypes of white spruce. These included the transcription factor *WOX2*, a member of the *WUSCHEL (WUS)*-related homeobox gene family of transcription factors whose expression is associated with specifying apical cell identity during the earliest stages of *Arabidopsis* embryogenesis (Mayer et al., 1998); *CHAP3A*, a spruce homolog of *LEAFY COTYLEDON1 (LEC1)*, a novel NF-YB subunit of the CCAAT-binding transcription factor that functions as a master regulator of embryogenesis (Lotan et al., 1998); *SAP2C*, a spruce homolog of *BABY BOOM (BBM)*, encoding an AP2/ERF responsive transcription factor involved in promoting cell proliferation and morphogenesis during embryogenesis (Boutilier et al., 2002); and *VP1*, a spruce ortholog of *Arabidopsis-VP1/ABSCISIC ACID INSENSITIVE (ABI)3* gene, encoding a B3-domain transcription factor involved in ABA signaling (Giraudat et al., 1992). These transcription factors were highly expressed in EMs from primordial shoots of 10-year-old somatic white spruce during the early stages of SE induction, allowing EMs to be distinguished from callus and other tissues in the cultured shoot buds (Klimaszewska et al., 2011). *LEC1* was also highly expressed in callus from shoot explants of adult *P. radiata* trees, raising the possibility that these lines had embryogenic characteristics (García-Mendiguren et al., 2015). In *Pinus contorta, PcWOX2* could be used as an early marker of EMs (Park et al., 2010). However, *P. contorta PcHAP3A* was expressed in all cell lines derived from bud cultures (embryogenic, non-embryogenic, and callus), indicating that it may be involved in cell division and could not be used as a marker of EMs like in white spruce (Klimaszewska et al., 2011). *PaVP1* expression was detectable only in embryogenic lines of *Picea abies*, and its expression increased strongly after transition onto maturation media containing ABA. Its expression was also decreased in a developmentally arrested line that could not form mature embryos (Fischerova et al., 2008). The *WOX* gene family was found to be associated with PEMs and EMs during early somatic embryo development in *Picea* (Palovaara and Hakman, 2008; Palovaara et al., 2010; Hedman et al., 2013; Li Q. et al., 2014). Klimaszewska et al. (2010) investigated the effects of 17-β-estradiol-induced ectopic expression of *A. thaliana WUS* (Zuo et al., 2002) and its spruce homolog *LEC1* on the development and growth of transgenic white spruce, early and late somatic embryos, intact somatic seedlings, and somatic seedling segments. *WUS* produced severe phenotypes when overexpressed, resulting from the disrupted development of somatic embryos on maturation medium and inhibition of germination. Neither of the transgenes induced ectopic SE, even in the presence of plant growth regulators, indicating that overexpression of *WUS* or *CHAP3A* could not maintain SE ability. A high expression level of the transgenes did not affect the expression profiles of genes encoding other transcription factors involved in embryogenesis. The relative transcript level of *BBM* was also higher during direct induction of embryogenic cell clusters than during direct induction of somatic embryos in Siberian ginseng. In addition, the expression levels of *PLETHORA* genes, encoding an AP2 transcription factor regulating auxin-depending root meristem identity, increased in direct embryogenic cell clusters during the culture period, indicating that these genes may be involved in the capacity for embryogenesis (Zhou et al., 2014). In Chinese fir, *SOMATIC EMBRYOGENESIS RECEPTOR KINASE (SERK)1-2* and *WOX13* genes, the former encoding a LRR receptor-like kinase involved in the induction of SE competence in culture (Schmidt et al., 1997), were predominantly expressed in PEMs, the transition to late-stage PEMs associated with stress-related responses, and pro-embryo transition (Zhou et al., 2017). The enhanced performance of larch PEMs exposed to hydrogen was associated with antioxidant defense signaling, and thus, with the regulation of redox homeostasis (Liu et al., 2016). Compared with controls, the hydrogen-treated larch PEMs also showed significant differential expression of *BBM* and *WOX*, which encode critical regulators of SE, and genes encoding endonucleases, exonucleases, cysteine proteases, aspartic proteases, and metacaspases, which are involved in programmed cell death (PCD). The large number of differentially expressed genes encoding cell cycle regulators, PCD-related proteins, and BBM and WOX transcription factors suggested that these proteins may play crucial roles in PEMs establishment and development in larch (Liu et al., 2016).

Previous studies have identified several transcription factors involved in embryonic programs and cell fate determination in meristems that show significant changes in expression at the early reorganization stage of adventitious root formation (Sánchez et al., 2007; Ricci et al., 2008; Solé et al., 2008; Vielba et al., 2011; Rigal et al., 2012; Trupiano et al., 2013; Abarca et al., 2014; Brunoni et al., 2014). WOX and BBM were found to be associated with AR in poplar and larch (Liu et al., 2014; Li K.P. et al., 2014). Abarca et al. (2014) detected relatively high *GRAS* mRNA levels in non-differentiated proliferating embryogenic cultures and during somatic embryo development in *P. radiata*. The transcript levels of genes encoding putative GRAS family transcription factors, *SCARECROW (SCR), PrSCR*, and *SCARECROW-LIKE (SCL) 6, PrSCL6*, were significantly reduced or non-existent in adult tissues that had lost the capacity to form adventitious roots, but were maintained or induced after the reprogramming of adult cells in rooting-competent tissues. Vielba et al. (2011) also described *GRAS* gene expression associated with the maturation-related decrease in adventitious root formation in chestnut. Other studies have reported asymmetrical increases in the transcript levels of other members of the *GRAS* gene family, *PrSCL1* (Sánchez et al., 2007; Vielba et al., 2011) and *PrSHR* (Solé et al., 2008), in the cambial region and rooting-competent cells of pine and chestnut. Such asymmetrical increases were not detected in non-competent cuttings, where a diffuse signal was detected, indicating that there is a specific and localized auxin-signal pathway in root progenitor cells (Sánchez et al., 2007; Solé et al., 2008; Vielba et al., 2011). Increases and asymmetrical distribution of *GRAS* mRNA levels were observed in rooting-competent cells of black walnut (Stevens et al., 2018). In this species, a diffuse signal was also detected in non-competent cuttings. Together, all of these results suggest that there is a degree of evolutionary conservation of this response among distantly related forest tree species.

The expression of genes coding for transcription factors involved in stem cell identity is a common feature during the induction of SE and organogenesis. Specific genes are induced by auxin or stress. In addition, high levels of mRNA have been measured before resuming cell division leading to SE or meristem formation. However, experimental data are insufficient to support the hypothesis of the maintenance of pre-existing pluripotent stem cells in the tissues. Auxin, stress and expression of stem cell identity regulators are interrelated during SE or meristem formation. The hierarchy of these pathways is unknown.

## Roles of Plant Cell Wall, Cytoskeleton, and Their Interactions

Modification of the growth direction involves interactions among the cell wall, plasma membrane, and cytoskeleton. Such interactions are required for the establishment of polarity and the induction or maintenance of new plant organs (Vilches-Barro and Maizel, 2015). Several studies have analyzed the role of the cell wall, the cytoskeleton, and their interactions on the capacity for SE. A temporary plasmolysis pretreatment using either mannitol or sucrose induced single and independent direct somatic embryos in Siberian ginseng, concomitant with a sharp increase in callose accumulation. Callose was deposited between the plasma membrane and cell wall of zygotic embryos and hypocotyl embryogenic cells after the plasmolysis treatment (You et al., 2006), and was required for SE in plasmolyzed explants (Tao et al., 2012). The authors hypothesized that the imposed interruption of cell-to-cell communication might physiologically isolate the cells, stimulating their reprogramming into embryogenic competent cells and inducing single embryo development. Transcripts of genes related to the cell wall and *LEA* genes significantly accumulated in embryogenic cell clusters of Siberian ginseng (Zhou et al., 2014). In the same species, genes encoding xyloglucan endotransglucosylases/hydrolases were highly expressed in embryogenic lines compared with non-embryogenic lines. The products of these genes are involved in xyloglucan remodeling and cell wall-loosening, which are required for cell expansion and proliferation (Zhou et al., 2014). These findings suggested that cell wall remodeling is crucial for the induction of embryogenic cell clusters in Siberian ginseng. Similarly, Aquea and Arce-Johnson (2008) found that genes encoding enzymes involved in pectin and galactomannan modification or degradation were differentially expressed between embryogenic and non-embryogenic cells of *P. radiata*, indicating that specific modifications in the cell wall composition may be important for SE. Enzymes participating in loosening and reorganization of the cell wall, such as xyloglucan endotransglucosylase/hydrolase, galactosidases, and pectinesterases were also found to be associated with the acquisition of embryogenic competence in oil palm and *Pinus nigra* (De Carvalho Silva et al., 2014; Klubicová et al., 2017). These enzymes were induced by hydrogen treatment, an antioxidant molecule, which increased the ratio of active pro-embryogenic cells and normal somatic embryos during SE in larch (Liu et al., 2016). Their encoding genes were also found to be up-regulated in the embryo-suspensor of Norway spruce (Reza et al., 2018).

To understand the molecular mechanisms regulating the role of the cell wall in the initiation of SE, Li Q. et al., 2014 performed a transcriptome analysis of embryogenic and non-embryogenic tissues of *Picea balfouriana* genotypes. They found that several genes encoding arabinogalactan proteins (AGPs) were upregulated in embryogenic tissues compared with non-embryogenic ones. This finding indicated that cell wall signaling and intercellular communication play important roles in the acquisition of embryogenic competence and the initiation of SE in this species. Early work on SE in Norway spruce described the importance of the interactions among cells, and the roles of cell wall signaling and chitinase-cleaved forms of AGPs for induction of SE (Egertsdotter and von Arnold, 1995). An increased abundance of extracellular AGPs was detected in a highly embryogenic *Abies* hybrid cell line, compared with low embryogenic lines. The AGPs progressively accumulated within the extracellular surface matrix network, a structure covering embryogenic cells, during the early stages of embryo development (Samaj et al., 2008). They were not detected at the surface of non-embryogenic suspensor cells and callus cells from both lines.

Cell wall remodeling by increasing pectin esterification and an increase in endogenous auxin occur concomitantly at the early stages of two embryogenesis pathways: microspore embryogenesis; and embryogenesis from immature zygotic embryos. These pathways can be induced by different *in vitro* protocols, and have similar early embryogenic markers in *Q. suber* (Rodríguez-Sanz et al., 2014). Similar markers were found during the initiation of leaf SE in *Q. alba* (Corredoira et al., 2017).

Microtubules and microfilaments are involved in cell polarity and the orientation of the cell division plane, cell wall development, intracellular transport processes, the mechanical properties of the plant cell and tissues, and how these tissues interact with the plasma membrane and the cell wall. Therefore, along with the cell wall, these structures may play important roles in the organization of new structures during regeneration. Microtubules and F-actin were both found to be important for embryogenesis in Norway spruce (Smertenko et al., 2003). Cytochalasins and latrunculins (Lat B) are anti-actin drugs that induce actin depolymerization and modify the orientation of actin filaments. Treatment of Norway spruce embryonic cultures with these drugs affected embryo pattern formation by inhibiting meristematic cells, suspensor differentiation, and embryo maturation (Schwarzerová et al., 2010; Vondráková et al., 2014). The microtubule-associated protein, MAP-65, bound only to organized microtubules in embryogenic lines, and not to cortical microtubules in a developmentally arrested cell line that was incapable of normal embryonic pattern formation (Smertenko et al., 2003). The authors suggested that this was a criterion for PEMs to progress into early embryogeny (Smertenko et al., 2003). Modification of the cytoskeleton may also be related to the accumulation of auxin required to induce embryogenesis and to embryo polarization. Both auxin accumulation and embryo polarization are associated with polar auxin transport and the polarization of auxin transporters, resulting in the change in auxin distribution required for proper somatic embryo induction and development. Profilin is a multifunctional protein that links the plasma membrane to the actin cytoskeleton, and plays roles in signal transduction. Profilin was found to accumulate during SE in larch, indicating that it may play an active role in cytoskeleton organization during somatic embryo development (Zhao et al., 2015).

Several studies have identified components of the molecular pathways underlying cell wall and cytoskeleton remodeling which are associated with adventitious root formation and the maturation-related decline in AR in pine and *Eucalyptus* (Díaz-Sala et al., 1997; Hutchison et al., 1999; Abu-Abied et al., 2014; Pizarro and Díaz-Sala, 2019). These authors described the coordinated developmental and auxin-dependent regulation of genes encoding expansin, actin, and several microtubule and microtubule-associated proteins in juvenile and adult cuttings of pine and *Eucalyptus*. The relevance of microtubule remodeling in the maturation-related decline of AR has been demonstrated using microtubule-disrupting drugs, which increased the rooting capacity of adult cuttings of *Eucalyptus grandis* (Abu-Abied et al., 2014).

Flexibility of the interactions between the cell wall, plasma membrane, and cytoskeleton is required to establish new cell polarity, reorient cell division planes, modify growth direction and induce a new morphogenic program. The components involved in these pathways are regulated by auxin, stress or cell identity regulators during induction of SE or organogenesis. In addition, modifications in the cell wall and cytoskeleton result in changes in cell polarity and the polarization of auxin transporters and auxin gradients.

## Conclusions and Future Prospects

**FIGURE 1:**
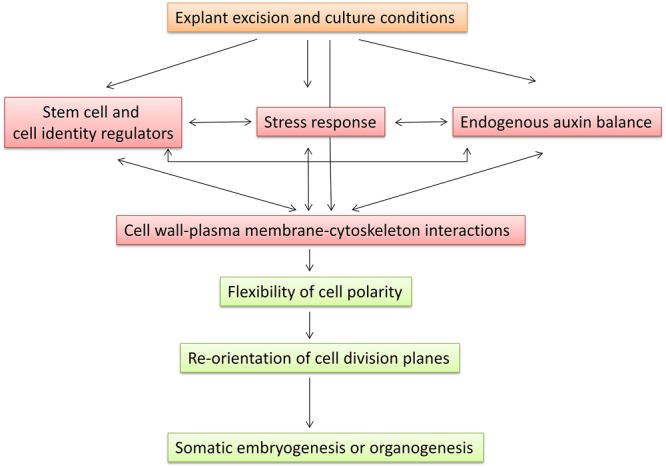
Proposed model for induction of somatic embryogenesis or organogenesis indicating common regulation pathways and their interaction.

Several interrelated pathways are involved in the plasticity of plant cells for SE or organogenesis in forest tree species (Figure 1 and Supplementary Table S1). Stress, auxin, and information carried by stem cell genes are common pathways associated with the competence of cells to initiate adventitious morphogenic programs. Stem cell genes were found to be related to the endogenous auxin gradient via the expression of auxin transport-related genes (Breuninger et al., 2008; Palovaara and Hakman, 2009). In addition, *SERK* was induced by auxin and stress conditions (Nolan et al., 2003; Santos and Aragão, 2009). Interestingly, two *PpWOX13*-like genes were found to be involved in the control of cell wall loosening to facilitate stem cell formation in *Physcomitrella patens* (Sakakibara et al., 2014). Crosstalk between microtubule remodeling and the cell wall has been found to be associated with PIN protein polarization, polar auxin transport, and auxin distribution, which are required for proper adventitious root formation in *Arabidopsis* (Abu-Abied et al., 2015a,b). All these results link the stress response, auxin gradients, stem cells, and cell wall remodeling, which may represent possible target pathways for developmental, environmental, or hormonal regulation of the regeneration capacity of forest tree species. The role of epigenetic regulation at different levels activating or rechanneling a developmental cell memory is an important mechanism regulating *de novo* regeneration. Molecular dissection of the mechanisms underlying regeneration capacity and identification of the genes expressed in common interrelated pathways regulating competence for SE and organogenesis would allow identification of an expressional signature characterizing specific levels of regulation, developmental stages, age, position, timing, type of tissues, clones or genetic traits associated with competence for SE or organogenesis. Cutting-edge technologies for analyzing multigene expression profiling may provide additional prognostic information for competence. Furthermore, the identification of rechanneling and master genes may provide additional tools for the modification of competence. Moving forward in the same direction, a more precise characterization of tissues used in operational programs will allow individualized management after diagnosis.

## Author Contributions

CD-S conceived the idea, gathered data, and wrote the manuscript.

## Conflict of Interest Statement

The author declares that the research was conducted in the absence of any commercial or financial relationships that could be construed as a potential conflict of interest.
